# Waking up the silenced beauty: CRISPR/Cas9 mediated reactivation of fetal hemoglobin genes to treat severe beta-thalassemia in young patients

**DOI:** 10.1093/lifemedi/lnad009

**Published:** 2023-03-06

**Authors:** Liren Wang, Stefan Siwko, Dali Li

**Affiliations:** Shanghai Frontiers Science Center of Genome Editing and Cell Therapy, Shanghai Key Laboratory of Regulatory Biology and School of Life Sciences, East China Normal University, Shanghai 200241, China; Institute of Biosciences and Technology, Texas A&M University, Houston, TX 77025, USA; Shanghai Frontiers Science Center of Genome Editing and Cell Therapy, Shanghai Key Laboratory of Regulatory Biology and School of Life Sciences, East China Normal University, Shanghai 200241, China

**Figure 1.**
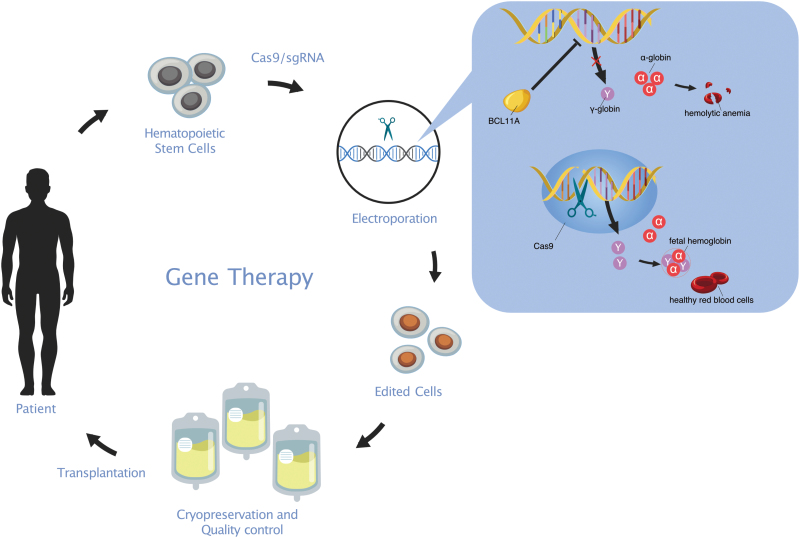
A schematic view depicting the clinical trial’s overall course and general molecular mechanism.

Since the birth of the CRISPR/Cas9 technology, the public has been expecting the realization of CRISPR-related therapies for hard-to-treat diseases, because there has never in the history of research been any tool that can manipulate genetic material with such ease, efficiency, and versatility. In the past 2 years, sporadic clinical results have shed light on CRISPR technology’s effectiveness and safety [1], encouraging researchers to invest more effort in these types of translational studies. In the recent issue of *Nature Medicine*, Bin et al. took a step further to treat the most severe kind of β-thalassemia (*β*^0^/*β*^0^) through CRISPR technology [2] (Fig. 1). The preliminary results from their ongoing phase 1/2 trial (NCT04211480) are promising. They demonstrated robust efficacy with the therapy, allowing two patients to be transfusion independent. Importantly, Bin et al. demonstrated that Cas9-mediated genomic editing did not create any off-target events above the baseline and did not affect the leukocyte progeny of the hematopoietic stem cells (HSPCs) for up to 18 months. How did Bin and his colleagues achieve their success? Where will this powerful genomic editing tool take us?

β-Thalassemia is one of the most common monogenetic diseases caused by abnormalities in the gene expressing the hemoglobin chain β (β-globin). Patients with β-thalassemia major have seriously compromised or no expression of β-globin, leading to insufficiency of adult hemoglobin (HbA) and toxic accumulation of free α-globin. These patients develop life-threatening hemolysis and anemia within two years after birth if they do not receive regular blood transfusions and other medical care. Currently, the only cure for β-thalassemia major is allogeneic HSPCs transplantation; however, over 80% of patients are ineligible for this option due to the shortage of immunologically matched donors. In China, β-thalassemia major patients tend to be younger due to limited blood resources and the high cost of iron chelators. This trend is also reflected in the two patients in this study, who were seven and eight years old, respectively. The genotypes of the two patients were *β*^+^/*β*^+^ (IVS2-654M) and *β*^0^/*β*^0^ (41-42M/17M), the latter of which presented a new challenge for Bin’s team because this would be the first time the treatment was performed on a patient whose β-globin was utterly absent.

Bin’s strategy is to phenotypically rescue the diseased HSPCs of the patients, turning them into autologous donor cells eligible for transplantation. Two patients were given G-CSF to mobilize their HSPCs, which were harvested by apheresis. To perform genomic editing, Bin’s team electroporated the Cas9:sgRNA ribonucleoprotein (RNP) into the purified HSPCs. Rather than fixing the mutant β-globin gene, their target was an enhancer region of the transcription factor BCL11A. BCL11A plays a crucial role in a process, namely the γ- to β-globin switch, which is critical for the onset of β-thalassemia. Studies have unveiled that β-thalassemia does not manifest during pregnancy or early infancy (before 6 months) because of the expression of fetal hemoglobin (HbF) consisting of the hemoglobin chain *α* and *γ*, the latter being the product of the HBG1/2 genes. Around 6 months postnatal, the HBG1/2 are silenced by a protein complex recruited by BCL11A; meanwhile, the β-globin expression is turned on to complete the γ- to β-globin switch. If an individual carries β-thalassemia alleles, he begins to manifest symptoms. By targeting the specific enhancer of BCL11A, Bin successfully turned BCL11A off in the erythroid lineages while maintaining its expression in the leukocytes. This lineage-specific “knockout” is vital for two reasons. First, in erythroid cells, the γ-globin expression is restored, acting to form HbF with α-globin, which phenotypically rescues the HSPCs (Fig. 1). Second, in leukocytes, the expression of BCL11A remains unchanged because it is indispensable for maintaining B cells and plasmacytoid DCs (pDCs). To achieve the best efficacy, the team confirmed the high initial editing frequencies for the two patients (patient 1, 97.17% and patient 2, 98.22%) before the edited HSPCs were cryopreserved. After 4 days of chemotherapy conditioning, two patients received their autologous transplantation.

For any clinical trial, patient safety is the priority. In this trial, the main adverse events (AEs) came from two aspects: engraftment-related AEs, and genomic editing-related AEs. The engraftment-related AEs included grade 4 neutropenia and thrombocytopenia, both of which were resolved as they are common in HSPC transplantation. The genomic editing-related AEs, mainly off-target cleavage, and the impact of Cas9 on the differentiation capability of HSPCs, are of great interest to the public. Bin et al. performed one of the most robust off-target detection assays—CIRCLE-seq, together with an in-silico prediction of off-target sites. Each potential off-target site was amplified from the edited cell pool and excluded by next-generation sequencing (NGS). Since HSPCs are ancestor cells for both erythroid cells and leukocytes, Bin et al. closely monitored the edited HSPCs from both patients. High editing frequencies were detected in multiple blood lineages, confirming normal differentiation capacity without malignant clonal expansion. Furthermore, through single-cell sequencing, they discovered that the transcriptomes of the edited PBMCs from both patients were within the normal range compared to healthy donors. This is an encouraging result for other CRISPR-related therapies since it demonstrated that the impact of the CRISPR protein could be limited to only the targeted gene by proper delivery methods and rational guide RNA design. With successful engraftment of edited HSPCs, γ-globin, and HbF gradually increased in the two patients, compensating for the function of the missing HbA. The two patients received their last blood transfusion on day 29 and day 19, respectively, and had been transfusion-independent for more than 18 months at the time their clinical trial was published.

In 2020, Frangoul et al. reported the clinical trial (NCT03655678) sponsored by CRISPR Therapeutics and Vertex Pharmaceuticals, treating a patient with sickle cell disease (SCD) and a patient with β-thalassemia major (*β*^+^/*β*^0^), in which a patient has traces of functioning β-globin left. In Bin’s trial, they pushed this strategy one step forward to treat the most severe type of β-thalassemia (*β*^0^/*β*^0^), proving its robust efficacy and safety in progeny cells. This is another milestone for treating genetic diseases in the “CRISPR way.”

Blood cells are ideal targets for researchers studying CRISPR-related therapies due to their ease of collection and *in vitro* culturing. Several biotech startups have commenced clinical trials targeting BCL11A or other sites for the treatment of SCD or β-thalassemia. In erythroid lineage cells, BCL11A binds to the promoter region of HBG1/2 gene to silence γ-globin. Instead of targeting BCL11A +58 enhancer to specifically turn off its expression in erythroid lineages as described in Bin and Frangoul’s reports. Editas, another famous CRISPR biotech, used the Cas9 homologues-Cas12a to disrupt the binding motif for BCL11A on the HBG1/2 promoter [3]. Previous results from cell culture and animal models have demonstrated a comparative efficacy by targeting the HBG1/2 promoter with CRISPR. However, detailed preliminary clinical data must be unveiled before we could systematically compare these strategies on patients. Meanwhile, Beam Therapeutics had developed two strategies to cure SCD using base editors, a CRISPR-based technology that introduces point mutations instead of generating double-stranded breaks (DSBs). Beam’s first strategy (NCT05456880) depends on the reactivation of γ-globin expression, not via Cas9 mediated DSBs, but via base editing in the HBG1/2 promotors to disrupt the BCL11A binding motif. Their second strategy functions by converting the sickle cell mutation into a benign variant by introducing an “A” to “G” transition into the HBB gene [4]. Since base editors generate almost no DSBs in the genome, theoretically, they will be safer alternatives to treat SCD or β-thalassemia. Except for reactivating γ-globin expression, some scientists are making efforts to seamlessly fix the diseased β-globin gene via CRISPR-stimulated homology-directed repair (HDR) [5]. Typically, HDR is very inefficient in cells; thereby, researchers devised an enrichment method by incorporating affinity tags into the donor sequence; they also harnessed other elements intended to increase HDR such as an AAV encapsulated single-stranded donor DNA template. Theoretically, both β-globin repair and γ-globin reactivation can be applied to treat β-thalassemia or SCD. Currently, the strategy of γ-globin reactivation proceeds much faster in clinical trials, for NHEJ is much more efficient than HDR and editing efficiency corresponds positively with efficacy. However, the advantage of HDR strategy is that it restores the wild type HBB gene, which mimicks the genotype of a healthy person. Notably, the potential significance of HDR strategy also extends to other genetic diseases such as severe combined immunodeficiency (SCID) that can only be cured by fixing the mutated genes or incorporating a healthy coding sequence (CDS) into the genome.

Globally, 10 preliminary clinical results have been reported from three clinical trials (NCT03655678, NCT04211480, NCT04925206) which used the CRISPR technology to target BCL11A +58 enhancer for the treatment of SCD or β-thalassemia. For the four patients with sickle cell diseases, no vaso-occlusive crises have been reported after the transplantation; for the six patients with β-thalassemia, they have been transfusion independent till now. Therefore, we expected a high potential cure rate for patients who are eligible for the treatment in the future. The remaining questions are about long-term safety and efficacy. Is there an individual cell with malignant off-target edits? How will long-term expression of γ-globin affect the patient? These and other questions will be answered in future follow-up studies. The clinical trials of β-thalassemia and SCD are just a hint of the power of CRISPR-related gene therapies. Since CRISPR is readily incorporated into many other mature bio-techniques we can envision that in the foreseeable future, there will be an explosion of CRISPR-related clinical applications that can cure genetic diseases, eliminate infection, or even put a stop to the progression of cancer.
